# Sleep Disturbances in the Prodromal Phase of Mood Episodes in Patients with Bipolar Disorder: A Replicated Single-case Design

**DOI:** 10.17505/jpor.2025.28202

**Published:** 2025-10-02

**Authors:** Stefan E. Knapen, Evelien Snippe, Arnout C. Smit, Robert A. Schoevers, Rixt F. Riemersma-van der Lek

**Affiliations:** 1 University of Groningen, University Medical Center Groningen, Department of Psychiatry, Interdisciplinary Center Psychopathology and Emotion regulation (ICPE).; 2 Leiden University Medical Center, Department of Neurology.; 3 University of Groningen, Department of Developmental Psychology.; 4 Mental Health Services Drenthe

**Keywords:** Bipolar disorder, chronobiology, actigraphy, sleep disturbances, experience sampling method, temporal relationship, time-series analysis

## Abstract

This study aims to examine whether objectively measured sleep disturbances occur in the prodromal phase of mood episodes in patients with bipolar disorder. Thirteen patients with bipolar disorder were studied using a replicated single-case time-series design for 180 days with continuous actigraphy and a daily Ecological Momentary Assessment of mood symptoms. Eight patients were suitable for analysis. Sleep variables (sleep onset, sleep offset, sleep efficiency, sleep duration, sleep onset latency, minutes awake after start of sleep, composite phase deviation) were estimated using actigraphy. Mean shifts and extreme values in the data were assessed using change point analysis and statistical process control. Mean shifts and extreme values in sleep were studied in the two weeks preceding depressive episodes and manic episodes. Changes in sleep were observed in the two weeks preceding mood episodes in two out of three individuals with a manic episode and in four out of five individuals with a depressive episode. There were individual differences in the type of sleep variables that showed change. However, these changes did not occur at a higher rate than during phases in which patients were stable. The order of change in sleep and EMA assessed mood could not be disentangled. The current study illustrates the heterogeneity of the type of sleep disturbances as assessed with actigraphy in the weeks before mood episodes.

## Introduction

According to the Global Burden of Disease, bipolar disorder is significantly associated with disability worldwide (Murray et al., 2012). Bipolar disorder is a chronic psychiatric disorder characterized by depressive episodes and manic episodes, with more or less stable phases in between (American Psychiatric Association, 2013). The main treatment focus is preventing such new mood episodes, especially as patients with more mood episodes experience a worse course of the disorder with more harmful consequences of the disease in daily life (Bond & Anderson, 2015; Peters et al., 2014). Psychoeducation, alongside pharmacological treatments, aims to decrease the number and the severity of episodes through finding early markers of an upcoming mood change. For instance, an action plan, such as the wellness recovery action plan, enables a patient to acknowledge changes in their own behavior and mood (Copeland, 2000; Perry et al., 1999). However, patients and clinicians often lack objective markers to signal upcoming mood episodes. A possible objectively assessable marker of an upcoming manic or depressive episode might be sleep disturbances.

Sleep alterations are clearly implicated in bipolar disorder, being one of the diagnostic criteria for both depressive and manic episodes (American Psychiatric Association, 2013; Harvey, 2008). Sleep disturbances are also shown in the euthymic phase of the disease, the period where patients show no severe mood symptoms (Geoffroy et al. 2014; Ng et al., 2015), and even before the onset of bipolar disorder (Pancheri et al., 2019; Ritter et al., 2015). Furthermore, studies showed in samples of patients with bipolar disorder that circadian rhythm was associated with mood episode recurrence (Ferrand et al., 2022) and shorter time to relapse (Takaesu et al., 2017). A more direct relationship between sleep and mood symptoms has been shown in patients with bipolar disorder as well; a systematic review showed that when patients were asked what preceded their onset of a mood episode, a median of 77% (range: 53% – 90%) of the patients reported sleep disturbances as an early symptom of a manic episode, making it the most prominent prodrome (early symptom of a disorder) for manic problems (Jackson et al., 2003). In contrast, a median of 24% (range: 17% - 57%) of the patients reported sleep disturbances as a prodrome for a depressive episode.

Objectively measured sleep has been implied as a potential marker for change in mood in patients with bipolar disorder in the late 70s and early 80s (Wehr et al., 1979; Wehr et al., 1982). These studies showed that sleep disturbances and mood changes happen at about the same time. A more recent study using actigraphy to assess sleep showed that fluctuations in sleep efficiency and later sleep onset predicted increased sadness the following day in patients with bipolar disorder, while fluctuations in sadness predicted later sleep onset that evening (Patapoff et al., 2022).

Although the association between sleep disturbances and mood episodes as well as symptoms have been studied in numerous ways in patients with bipolar disorder (see Ulrichsen et al. [2025a] for a review), previous studies did not investigate whether sleep disturbances increase in the period just preceding a mood episode and whether these sleep disturbances occur earlier than mood symptoms increase. One recent study examined the phase just preceding mood episodes and showed that late prodromal phases before depressive episodes and manic episodes could not be differentiated from euthymia based on self-reported sleep duration or time before falling asleep (Ulrichsen et al., 2025b). Only fewer hours spent sleepless in bed was associated with a higher chance of being in the late prodromal phase (Ulrichsen et al., 2025b).

To examine whether changes in objective sleep parameters precede mood episodes and mood symptoms, objective and non-invasive measurement of sleep and frequent mood assessment in daily life are needed. Both sleep and mood need to be monitored over a long period of time that includes both a mood episode as well as a period preceding the episode. The method of studying patient behaviour in real time and in their own setting is called Ambulatory Assessment (Trull & Ebner-Priemer, 2013) and provides a unique opportunity to study the link between sleep disturbances and mood symptoms. Mood symptoms can be monitored using daily diary momentary self-reports, as is done in Ecological Momentary Assessment (EMA) studies (Stone & Shiffman, 1994). A non-invasive way to study sleep objectively is using a wrist-worn device, a so called actiwatch, measuring activity over the day and night (Ancoli-Israel et al., 2003; Hofstra & de Weerd, 2008). Actigraphy has been validated with the golden standard of sleep, polysomnography, in bipolar disorder and is proven to be a cost-effective method to assess sleep in patients in their everyday life (Kaplan et al., 2012). A case report (Knapen et al., 2016) illustrated how such objective measures of sleep disturbances may inform clinical decision-making aimed at preventing psychopathology. In this case report, 1 mg of lorazepam was prescribed after sleep as measured with actigraphy indicated a later sleep onset and a shorter sleep duration (Knapen et al., 2016).

To study sleep disturbances and mood symptoms in the phase just preceding a mood episode in patients with bipolar disorder, we monitored 13 patients for 180 days, using both objective sleep measures and ecological momentary assessments of mood symptoms. Using a replicated single-case time-series design, we aimed to study whether extreme values in sleep measures (e.g., one extreme short sleep duration) and mean shifts in sleep measures (e.g., a systematic shift towards shorter sleep duration) would occur in the two weeks (i.e., the prodromal phase) before a mood episode. We expected the following sleep disturbances: increases in sleep onset, sleep onset latency, minutes awake after going to bed, composite phase deviation and decreases in sleep offset, sleep efficiency, and sleep duration. Increases in sleep offset and sleep duration can also be regarded as sleep disturbances. A second aim was to examine whether we could disentangle the temporal order in which sleep variables and mood symptoms change. We expected sleep variables to change in the two weeks before the mood episodes and mood symptoms around the start of the mood episodes.

## Methods

### Participants

Patients with bipolar disorder were recruited at the outpatient clinic of the University Center Psychiatry of the University Medical Center Groningen. Furthermore, patients were recruited at the Netherlands bipolar patients and carers organization (Plusminus).

Inclusion criteria were bipolar disorder type I and willingness as well as ability to wear an actigraph and complete a mood symptom diary daily for 180 days. Exclusion criteria were somatic diseases which could hamper actigraphy measures, and somatic sleep disorders (such as sleep apnea). Bipolar disorder type I and comorbid psychiatric disorders were confirmed using the Mini-International Neuropsychiatric Interview (MINI) during a baseline interview (Sheehan et al., 1998). Chronotype was assessed using the Munich Chronotype Questionnaire (Roenneberg et al., 2003). All patients provided written informed consent. The medical ethical committee of the University Medical Center Groningen waived the necessity to review the study because it decided the study did not fall under the Medical Research Involving Human Subjects Act (WMO) due to its non-invasive nature and as it was an extension of care as usual. All procedures were in accordance with the declaration of Helsinki (2013) and the Medical Research Involving Human Subjects Act.

Fifteen patients provided written informed consent for the study, of which one (participant #14) dropped out during the study because of personal reasons. Data from one participant (participant #5) was largely incomplete after the study and were excluded from the analysis as well. In total data from 13 patients were suitable prior to the analyses.

### Data acquisition

Sociodemographic variables, medication use, and health problems were assessed using a questionnaire. Patients participated in the study for 180 consecutive days. Participants received a text message or an e-mail with a link to a secured online environment where the questionnaire was filled in. This procedure was followed for both daily and weekly questionnaires.

### Measures

#### Sleep

Patients were instructed to continuously wear the Motionwatch 8 (CamNtech; a light weight, water-proof accelerometer), only taking it off when absolutely necessary (e.g., during a sauna visit). Every morning, patients completed an electronic sleep diary reporting when they turned the lights off the evening before, when they tried to fall asleep and how many minutes it took to fall asleep. They also reported at what time they got up and how they rated their own night. Sleep variables were calculated from the actigraphy data, using our own developed script in R. The script for the analysis can be retrieved from https://github.com/compsy/ACTman/ (Kunkels et al., 2020; R Core Team, 2018). The functionality of the script is to select a rest period where sleep is possible using the sleep log. From the activity during that period likely wake bouts are estimated. After the rest period was defined, the sleep algorithm was used to estimate sleep duration (the time between sleep onset and sleep offset), timing of sleep onset (the moment the subject fell asleep), timing of sleep offset (the moment the subject woke up), sleep onset latency (difference between bed time and the timing of sleep onset), sleep efficiency (i.e., minutes asleep divided by minutes in bed), wake after sleep onset (WASO, in minutes, the minutes wake bouts are scored, between the sleep onset and sleep offset timing). As a marker of circadian misalignment the *composite phase delay* was computed, which is the distance of the midpoint of sleep of the current night to the past night and to the ideal midpoint of sleep based on the chronotype of a subject (Fischer et al., 2016).

#### Mood symptoms

In the evening, patients completed the electronic Lifechart (LC-self), reporting their mood symptoms on a single VAS scale from “depressed” (0) to “manic” (100) (Born et al., 2014). Furthermore, they answered a set of 10-16 items every evening measuring positive and negative mood symptoms on a VAS scale ranging from “Not at all” to “Very much”. Such questions included “I feel down”, “I feel excited” and “I feel satisfied”. A full list of the mood symptom items can be found in Appendix A. Ten questions were mandatory to fill in, covering both an elevated and a decreased mood. Patients could select three additional “mania” items, and two additional “depression” items, and they could provide one question themselves as well.

#### Manic and depressive episodes

Manic and depressive episodes were defined using the Inventory of Depressive Symptomatology – Self Rating (IDSSR) and the Altman Self Rating Scale for Manic symptoms (ASRM) (Altman et al., 1997; Rush et al., 1996). Participants completed these questionnaires on a weekly basis during the study period. For a manic episode, patients had to score above five points on the ASRM on two consecutive weeks, making sure there was at least one full week of manic symptoms (Altman et al., 1997). Furthermore, patients had to report on the LC-self above the midline (i.e., higher than 50 on a scale ranging from “depressed” (0) to “manic” (100)) at least 75% of the time during the weeks that they scored above five points on the ASRM. For a depressive episode, patients had to score above 25 points on the IDS-SR on at least three consecutive weeks, resulting in at least two full consecutive weeks of symptoms. They also had to report their daily mood below the midline (i.e., lower than 50 on a scale ranging from “depressed” (0) to “manic” (100)) at least 75% of the time during the weeks that they scored above 25 points on the IDS-SR.

#### Prodromal phase and start of the episode

The systematic review of Jackson et al. (2003) found that the mean length of the prodromal phase of manic and depressive episodes was about three weeks. Therefore, we chose a period of three weeks before the mood episode was identified with the ASRM or IDS-SR as the focus of our study. Since the ASRM and IDS-SR were used to assess symptoms during the past week, the week preceding the identification of the mood episode was regarded as the week in which the episode started. The two weeks before the week in which the episode started was regarded as the prodromal phase.

### Statistical analyses

#### Extreme values

Each time-series of each mood symptom item and each sleep variable were analyzed for each individual separately. Extreme values were quantified using Shewhart control charts (Montgomery, 2009; Benneyan et al. 2003). First, a stable period before the manic or depressive episode was selected in order to define the extreme values. Stable periods were defined as five consecutive weeks of no manic or depressive symptoms (i.e., no ASRM > 5 or IDS-SR > 25 for at least two weeks) and a lower variability on the Life Chart compared to the full 180 days. This five-week period was selected to reflect 35 days, which is similar to a previously found ideal period to detect special-cause variation (Wiemken et al., 2017). The stable periods were selected as early as possible in the data and had to end at least two weeks before the onset of an episode. The first and the last author performed all selections. Patients were excluded when no stable period of five weeks before the mood episode could be selected. Using data of the stable period, we computed the mean of each variable of interest and included upper and lower limits which consisted of three times the standard deviation of that variable during the stable period. Whenever a variable scored out of these limits, this was marked as an extreme value.

#### Mean shifts

To detect structural mean shifts in the sleep and mood variables, changepoint analysis was performed using the *breakpoints* function of the *strucchange* package in R. This function estimates when a significant structural change in the time series occurred (Zeileis et al., 2002). For every variable (both mood items and sleep variables) the change point and confidence intervals around the change point were estimated using the sequential *F*-test from the *strucchange* package in R (Zeileis et al., 2002, 2003), requiring a minimal segment size of 7 days. The period from the start of the stable date up until the end of the episode period was used for the changepoint analysis.

#### Sleep disturbances in the prodromal phase preceding mood episodes

**Figure 1:**
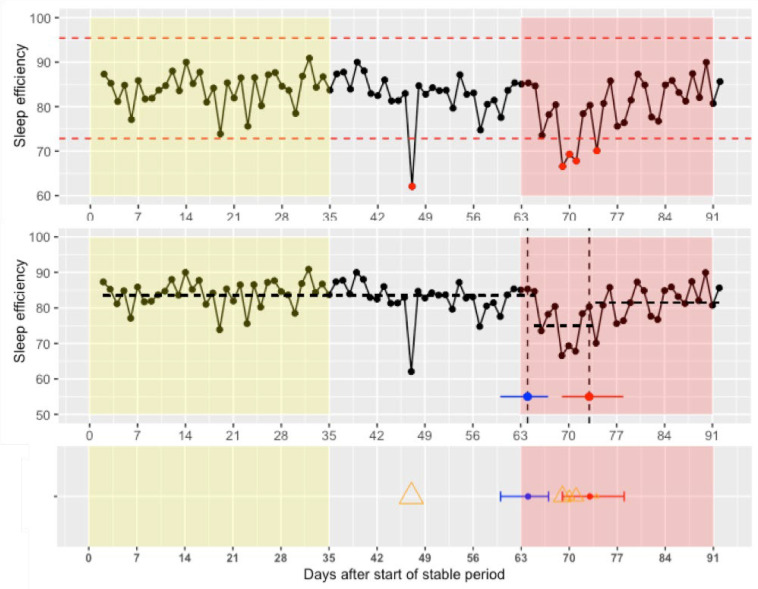
Example of detection of mean shifts and extreme values based on raw data *Note:* The upper panel shows the Shewhart control chart of participant 1 (randomly chosen), where the dashed lines show the upper and lower limit as calculated by 3 times the standard deviation of the variable in the stable period (marked as yellow). Point above or below those limits are extreme values marked red and transferred as triangles to the lower panel. The size of the triangle is based on how much the extreme values deviate from the mean. The middle panel presents the results of the change point analysis, where the black dashed horizontal lines show the mean of that the variable during that period, the vertical black dashed lines and the red and blue dot show the change points, and the horizontal blue and red lines represent the confidence intervals. Change points and confidence intervals are transferred to the lower panel as red and blue dots with confidence intervals. For each variable, we inspected whether change in the sleep variables occurred in the two weeks preceding the mood episode (i.e., 14 days before the red bar). In this case, no change was found in the prodromal phase (i.e., the 14 days before the mood episode) and decrease in sleep efficiency (blue dot) was observed around the start of the mood episode.

All extreme values from the Shewhart control charts and change points (CPs) from the change points analysis were exported and plotted over time (see Figure 1 for an example). In the plot, the stable period (yellow bar), prodromal phase before the mood episode (two weeks before the red bar), and the mood episode (red bar for depressive episode and green bar for a manic episode) are depicted. The first week of the green or red bar is the week in which the episode started. We studied which sleep variables showed mean shifts or extreme values in the two weeks before that in each patient individually. It is reported whether the variables increased, decreased, were unstable, or showed no change. Change was marked as unstable if both an increase and a decrease were found in a specific variable using the CP analysis or the Shewart control chart.

#### Temporal order of change in sleep variables and mood symptoms

**Figure 2:**
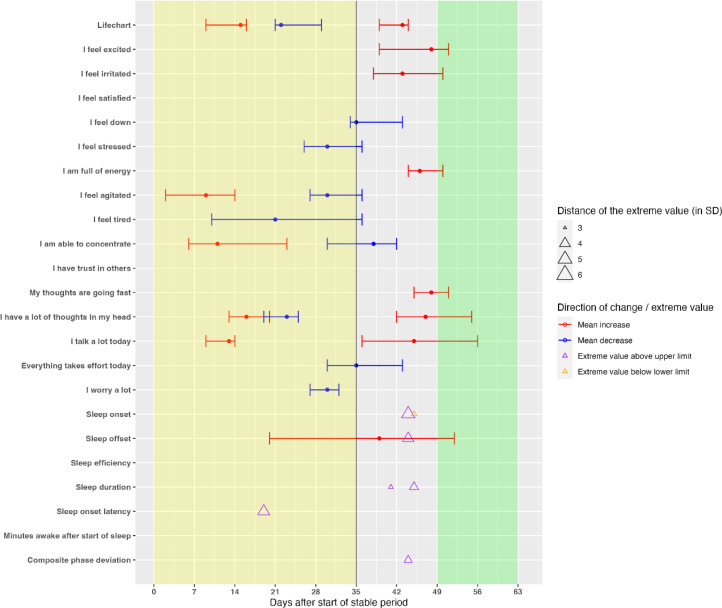
The selection of mean shifts and extreme values in one patient. *Note:* For both the sleep variables and mood symptoms it was determined whether they changed in the two weeks before the mood episode (i.e., the prodromal phase), that is, between the black line and the green bar (i.e., the manic episode) or during the first week of the mood episode (the first seven days of the green bar). In this case, both changes in sleep variables and mood symptoms occurred during the two weeks preceding the manic episode. Next, the confidence intervals of these selected variables were used to determine whether sleep symptoms preceded or followed on the mood symptoms or whether there was no significant difference in the moment of change. In this case, the sleep changes were all within in the confidence interval of the changes in mood symptoms so there was no significant difference in the moment sleep variables and mood symptoms changed.

Next, we examined whether we could disentangle the temporal order in which sleep variables and mood symptoms change in the prodromal phase preceding a mood episode. We examined whether change in the sleep variables preceded change in the mood symptoms by examining their timing and relative order during the two weeks preceding the mood episodes and the week of the start of the mood episode. This process was repeated for all variables, resulting in one large CP/outlier plot for each individual (Figure 2). When the confidence intervals of one or more sleep variables overlapped with the confidence intervals of one or more mood symptoms, we concluded that no specific order could be determined.

## Results

### Group characteristics

Of the 13 patients included in the study, 11 patients experienced a mood episode during the study period. Of these 11 patients, 3 did not have a “stable” period of five weeks in the period before the mood episode and were therefore excluded from the analyses. This resulted in 8 patients suitable for analysis. Patients were aged between 31 and 67 years. Seven patients were women and one was man. As mood stabilizer, five participants used lithium, one participant used carbamazepine, one participant used valproate acid, and one participant used lamotrigine. One participant used olanzapine and escitalopram, another participant used olanzapine and fluoxetine, and one participant used venlafaxine. The median mood episode duration was 3 weeks (range 2 to 4 weeks). Of the eight patients with a mood episode, three experienced a manic episode and five a depressive episode. The weekly scores, together with the highlighted selection of stable and episode periods for all individuals are presented in Appendix B (Figures B1b-B8b).

### Sleep disturbances in the prodromal phase preceding a mood episode

Figure 1 shows the data of one participant to illustrate how we determined whether sleep disturbances occurred in the prodromal phase preceding a mood episode. Of the three participants with a manic episode, we found in two participants changes in the sleep variables in the prodromal phase (see Table 1). Participant 8 had one night with an increase in sleep onset, sleep offset, and composite phase deviation, followed by a night with a decrease in sleep onset and an increase in sleep duration in the week anticipating the manic episode. This participant also had a mean increase in sleep offset during the prodromal phase. Participant 4 had one night with a decrease in sleep onset and another night with a decrease in sleep duration. Changes in sleep efficiency, sleep onset latency, and minutes awake were not observed preceding a manic episode. Sleep disturbances were not unique to the prodromal phase as they were also observed during the stable period (see Appendix B, figures B1a-B8a).

**Table 1:** Sleep changes in the prodromal phase preceding mood episodes.

	Manic episode				Depressive episode			
	Participant 2	Participant 4	Participant 8	Participant 1	Partcipant 3	Participant 9	Participant 11	Participant 15
**Sleep onset**								
** Mean shift(s)**	-	-	-	-	-	-	-	-
** Extreme value(s)**	-	Decrease	Unstable	-	-	Decrease	-	-
**Sleep offset**								
** Mean shift(s)**	-	-	Increase	-	-	-	-	-
** Extreme value(s)**	-	-	Increase	-	-	-	-	-
**Sleep efficiency**								
** Mean shift(s)**	-	-	-	-	Increase	-	-	Decrease
** Extreme value(s)**	-	-	-	-	-	-	-	-
**Sleep duration**								
** Mean shift(s)**	-	-	-	Increase	-	-	-	-
** Extreme value(s)**	-	Decrease	Increase	-	Decrease	-	-	-
**Sleep onset latency**								
** Mean shift(s)**	-	-	-	-	-	-	-	-
** Extreme value(s)**	-	-	-	-	-	-	-	Increase
**Minutes awake**								
** Mean shift(s)**	-	-	-	-	Decrease	-	-	-
** Extreme value(s)**	-	-	-	Increase	Increase	Increase	-	-
**Composite phase deviation**								
** Mean shift(s)**	-	-	-	-	-	-	-	-
** Extreme value(s)**	-	-	Increase	-	-	-	-	-

*Note:* Mean shift(s) are changes as indicated by the change point analysis. Extreme value(s) are changes as indicated by the Shewhart control charts. An increase or decrease may be based on one or multiple changes.

In four of the five participants with a depressive episode, we found changes in at least one of the sleep variables in the prodromal phase (see Table 1). Participant 1 had an increase in sleep duration and one night with an increase in minutes awake after start of sleep. Participant 3 showed an increase in sleep efficiency, one night with a shorter sleep duration, as well as a decrease in minutes awake after start of sleep and one night with an increase in minutes awake. Participant 9 had one night with a decrease in sleep onset, and one night with an increase in minutes awake after start of sleep. Participant 15 had a decrease in sleep efficiency and one night with an increase in sleep onset latency. Changes in sleep offset and composite phase deviation were not observed preceding a depressive episode. Sleep disturbances were not unique to the prodromal phase as they were also observed during the stable period (see Appendix B, figures B1b-B8b).

### Temporal order of change in sleep variables and mood symptoms

Next, we examined whether mean shifts in mood symptoms occurred after mean shifts and extreme values in the sleep variables, or whether the moment of change did not differ significantly (see Figure 2 and Table 1). For all three participants with a manic episode, the moment of change in mood symptoms and change in sleep variables did not significantly differ (i.e., the moment of change in sleep overlapped with the confidence interval of the change in mood). Participants 4 and 8 showed changes in both mood symptoms and sleep variables in the two weeks preceding the manic episode. Participant 2 had changes in both mood symptoms and sleep variables around the start of the manic episode.

For all participants with a depressive episode, the moment of change in mood symptoms and sleep variables did not significantly differ. Participant 1 and 3 showed change in mood symptoms and sleep variables both during the two weeks preceding the depressive episode and around the start of the depressive episode. Participant 9 showed mainly changes in sleep and mood during the two weeks preceding the episode, and only a few around the start of the episode. Participant 11 only showed changes in mood symptoms in the two weeks preceding the depressive episode. Participant 15 showed changes in mood symptoms and sleep variables only during the two weeks preceding the depressive episode.

## Discussion

In this paper, we set out with the aim to examine whether sleep disturbances as assessed using actigraphy occur in the prodromal phase (i.e., two weeks) preceding mood episodes in patients with bipolar disorder. Our second aim was to investigate whether changes in sleep variables preceded changes in mood symptoms as assessed with EMA. In the eight patients with a mood episode suitable for analysis, we found that two out of three manic episodes were preceded by sleep disturbances and that four out of five depressive episodes were preceded by sleep disturbances. There were individual differences in the type of sleep variables that changed in the prodromal phase. Furthermore, sleep disturbances did not occur more often in the two weeks preceding the mood episodes or around the start of the mood episode compared to periods in which patients were relatively stable. We therefore cannot conclude that sleep disturbances are reliable early markers of an upcoming mood episode. Furthermore, we could not disentangle the temporal order of change in sleep and mood symptoms.

The current study illustrates the heterogeneity of changes in sleep in the prodromal phase of mood episodes in individuals with a bipolar disorder. Not one of the participants showed exactly the same changes in sleep variables preceding mood episodes. Some participants differed in the direction of the change. For example, one participant showed an increase in sleep efficiency while two others had a decrease in sleep efficiency preceding a depressive episode. These findings show individual differences in the type of disturbances in sleep occurring preceding mood episodes, which implies that clinicians and patients should first explore patients’ individual patterns.

We could not disentangle the temporal order in which sleep and mood symptoms change. Changes in both sleep variables and mood symptoms were observed for most individuals during the prodromal phase. In some individuals, changes in mood and sleep variables were observed in the week that the mood episode started. The changes in sleep variables occurred very close to the mood symptoms change, which resulted in overlapping confidence intervals. This could imply that changes in sleep and mood follow one another so quickly that their order cannot be established with the current method. In line with this, studies focusing on daily fluctuations in mood and sleep using diaries showed that sleep duration was significantly decreased in the nights prior to next day increases in mood in the manic direction (Bauer et al., 2006; Bauer et al., 2008; Leibenluft et al., 1996). Furthermore, a study showed that fluctuations in sleep efficiency and later sleep onset predicted next day sadness and that increases in sleep were followed by next day increases in depressive mood (Patapoff et al., 2022).

Since we found changes in sleep variables both in the prodromal phase and during more stable periods, it might as well be that the found changes represent merely natural variability in sleep occurring in patients with bipolar disorder. This aligns with findings that sleep disturbances also occur during euthymic states (Geoffrey et al., 2015; Ng et al., 2015) and a study finding few differences in self-reported sleep parameters between late prodromal phases and euthymic phases (Ulrichsen et al., 2025b). The latter study indicated that variability in sleep parameters may be a more promising variable to distinguish late prodromal phases and euthymic phases in patients with bipolar disorder (Ulrichsen et al., 2025b). Another explanation for our findings is that we used a very restrictive method for examining whether the moment of change in mood and sleep differed by examining whether changes in sleep fell outside the confidence intervals of changes in mood. Furthermore, the fact that we investigated multiple sleep variables and mood symptoms increases the chance that the moment of change of some of the variables overlap. Finally, some of the mood symptoms, such as worrying and stress, may also be expected to occur already in the prodromal phase of a mood episode.

Future studies may observe individuals with bipolar disorder for a longer period of time in which multiple episodes take place and examine whether the same sleep disturbances precede these different episodes within the same individual. Furthermore, future studies may focus on examining analyzing actigraphy data in real-time, so that change in sleep variables can be detected during the data collection. This is especially interesting since commercial activity trackers become more advanced and more readily available (Blaauw et al., 2015). Specifically in patients with bipolar disorder this is promising, as patients often show an interest in applications for self-management of their disease (Nicholas et al., 2017).

To study the temporal order of change, we used an innovative approach by applying statistical process control and change point analysis to actigraphy data in the period just preceding mood episodes. This study is one of the few studies studying objectively assessed sleep variables specifically in the phase preceding a mood episode. One other study examined change points in objectively assessed sleep variables in the phase preceding and following recurrence of depression in patients with unipolar depression (Minaeva et al., 2024). Although patients have been studied in the clinic for a longer time, studying patients in their own daily life with objective measures is relatively new. Another strength of the study is that we used a replicated single subject timeseries design, ensuring that we revealed knowledge about processes occurring within individuals over time.

However, there are some limitations to this paper. Most notably is the small sample size with only eight patients suitable for analysis. This implies that with another sample, other changes in sleep variables may be found in the prodromal phase, also taking into account the heterogeneity we found in the type of changes preceding mood episodes. Another limitation of the study is that the analysis plan of the current study was not pre-registered. In addition, we did not have contextual information on circumstances that may have explained the sleep disturbances, such as a later sleep onset because of a birthday party or lower sleep efficiency because of an ill child. Sleep disturbances because of such circumstances may not be related to the onset of a manic or depressive episode. A further limitation is the fact that we mainly studied sleep variables, while it is also possible to calculate circadian rhythm variables from actigraphy data. As changes in the rhythm might be an interesting early warning signal itself as well, this might be a promising extension of this current work (Novak et al., 2014; Takaesu et al., 2017). However, the methods we used are specifically suitable for variables assessed on a daily basis and the circadian rhythm variables are typically calculated over a period of at least a week (van Someren, 2007). In order to still retain some circadian rhythmicity variables in the data, a variable which can be calculated on a daily basis, the *composite phase delay* was computed and used in the analyses as well (Fischer et al., 2016). Finally, the study only provides insight in the two weeks preceding the start of a mood episode, while the prodromal phase of some individuals may have been longer.

In conclusion, we used an innovative approach to study sleep disturbances in the prodromal phase preceding mood episodes in patients with bipolar disorder. Although sleep disturbances were observed in the two weeks preceding mood episodes, they did not occur more often than during phases in which participants were relatively stable. The current study illustrates the heterogeneity of changes in sleep occurring in the period before mood episodes. We could not disentangle the temporal order of changes in sleep as assessed with actigraphy and changes in mood symptoms as assessed with EMA.

## Figures and Tables

**Figure B1a:**
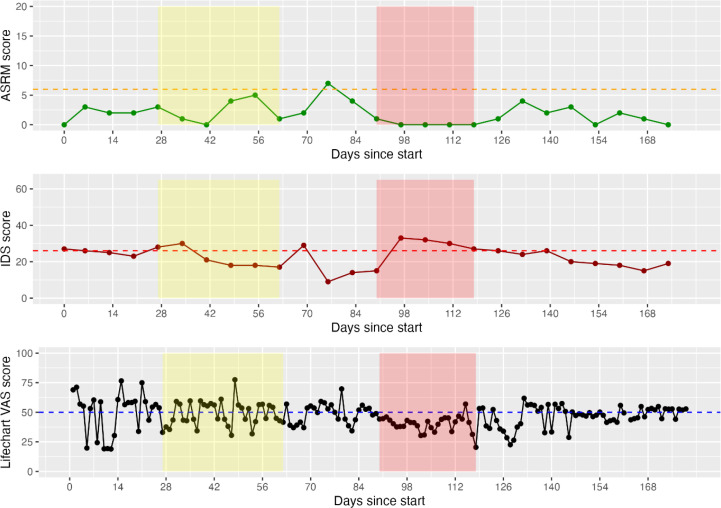
Weekly scores of subject 1

**Figure B1b:**
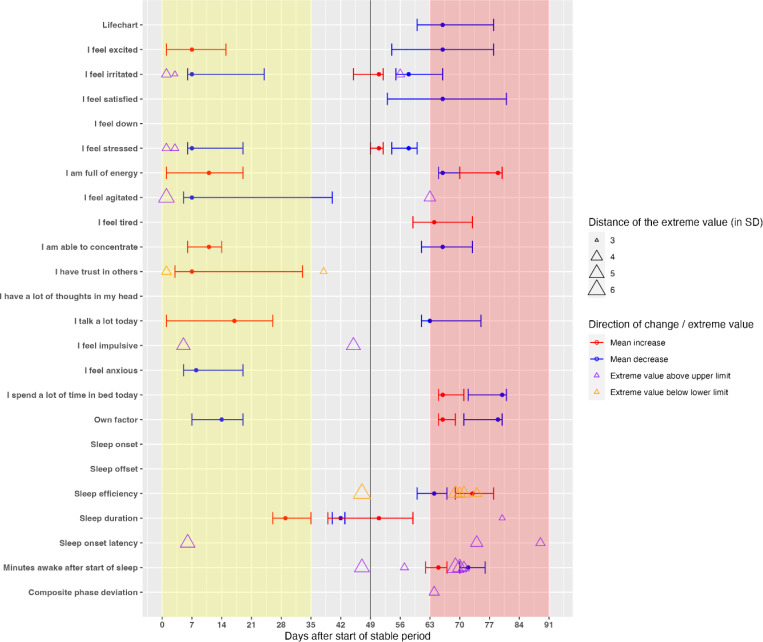
Change points and extreme values of subject 1

**Figure B2a:**
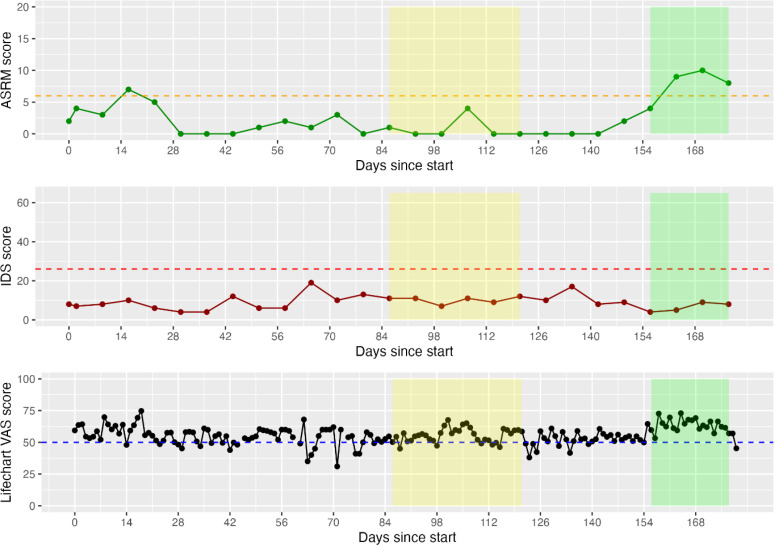
Weekly scores of subject 2

**Figure B2b:**
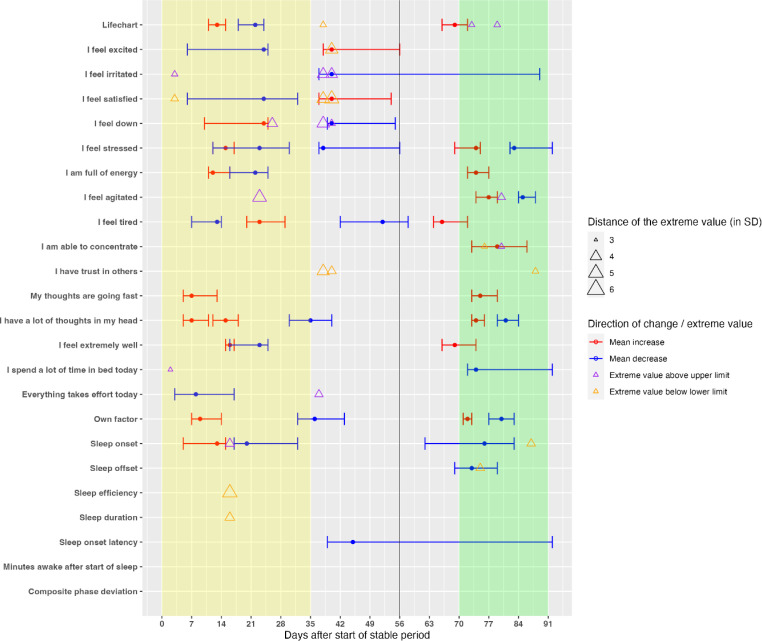
Change points and extreme values of subject 2

**Figure B3a:**
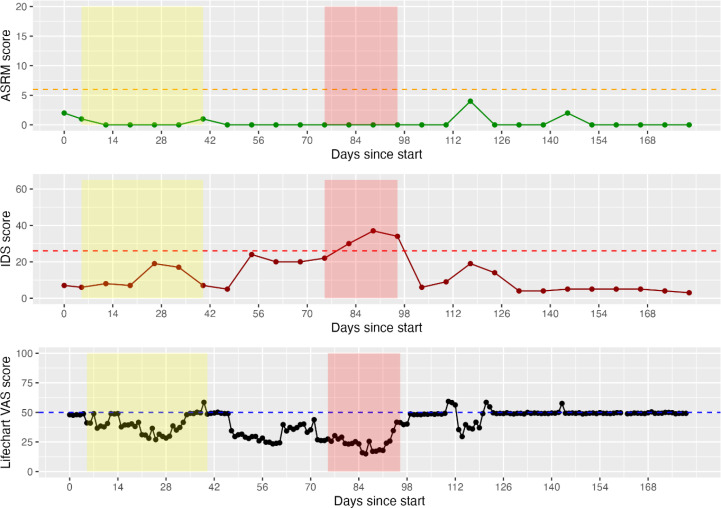
Weekly scores of subject 3

**Figure B3b:**
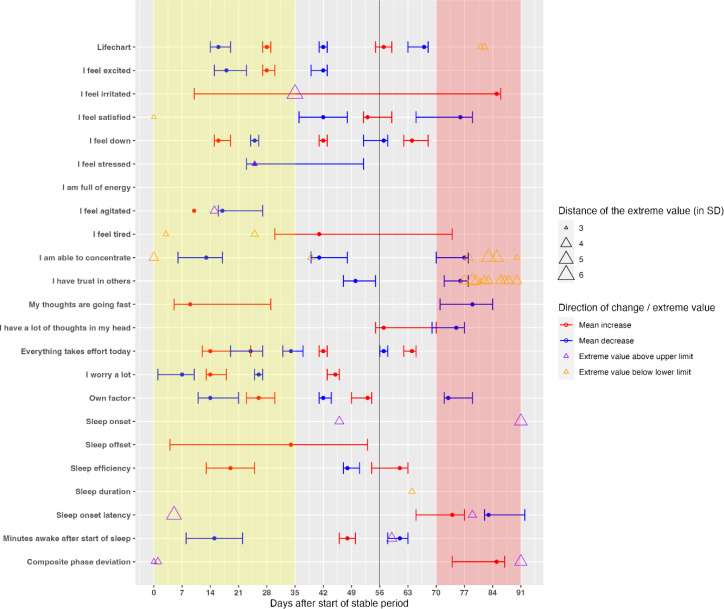
Change points and extreme values of subject 3

**Figure B4a:**
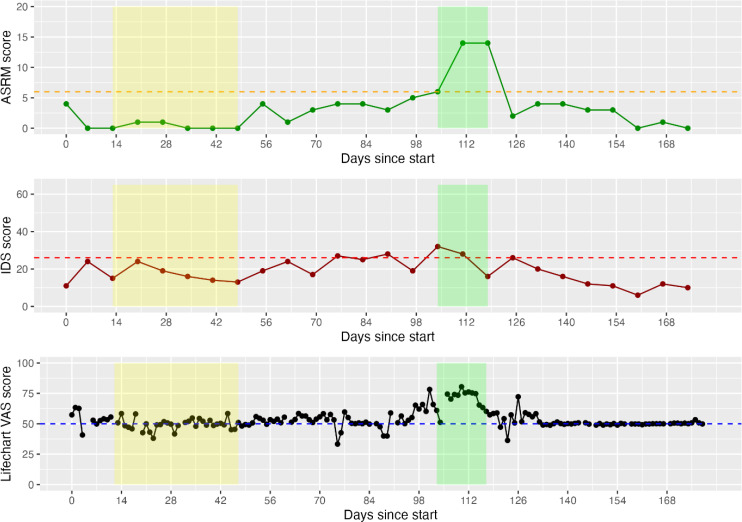
Weekly scores of subject 4

**Figure B4b:**
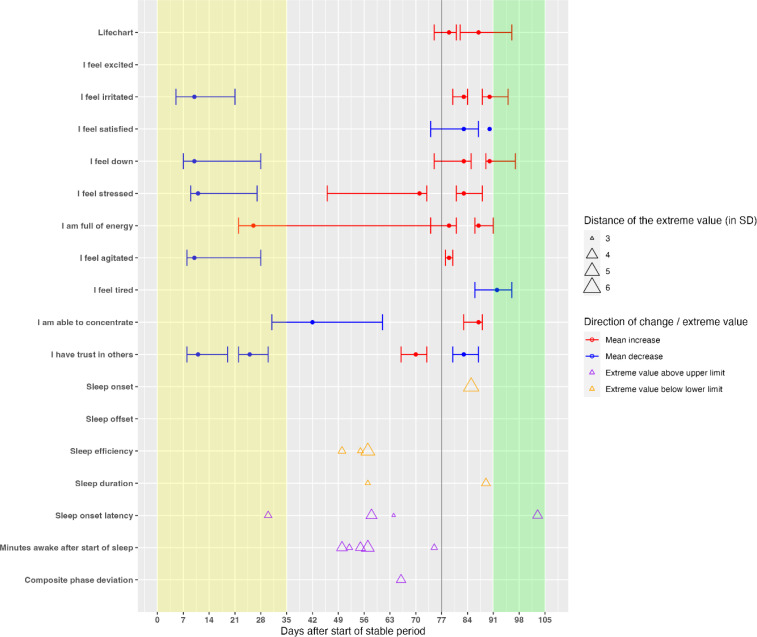
Change points and extreme values of subject 4

**Figure B5a:**
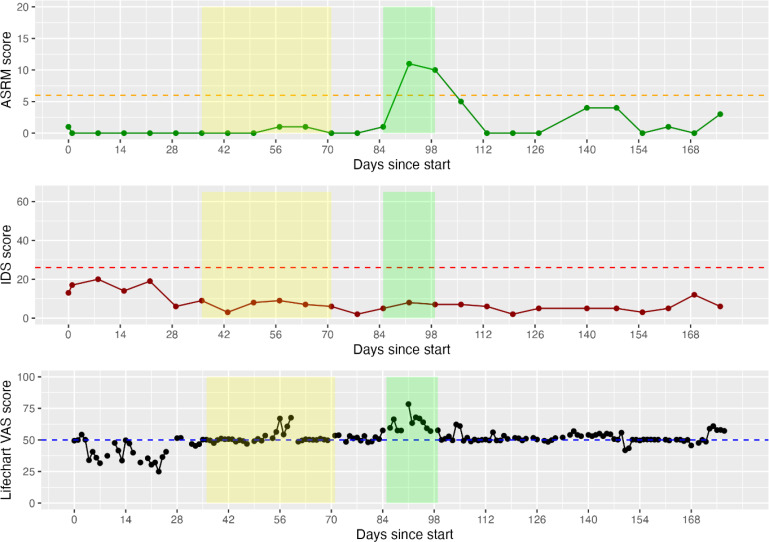
Weekly scores of subject 8

**Figure B5b:**
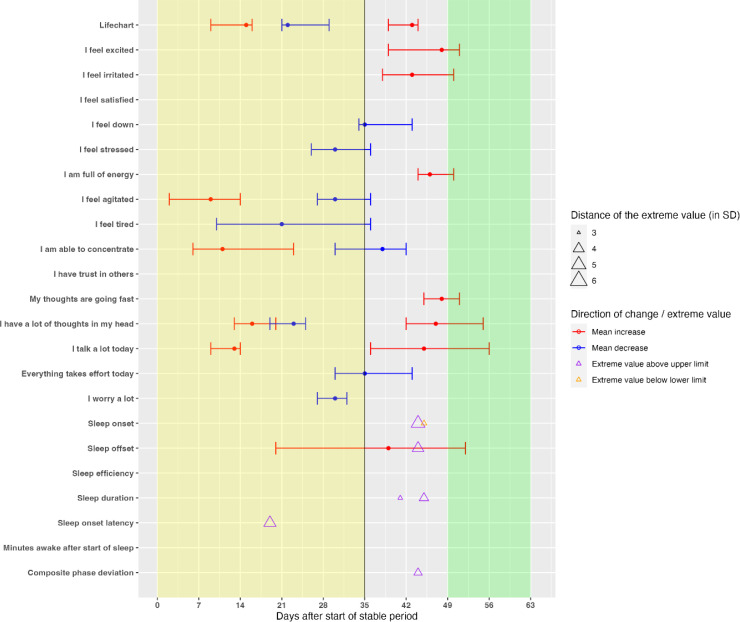
Change points and extreme values of subject 8

**Figure B6a:**
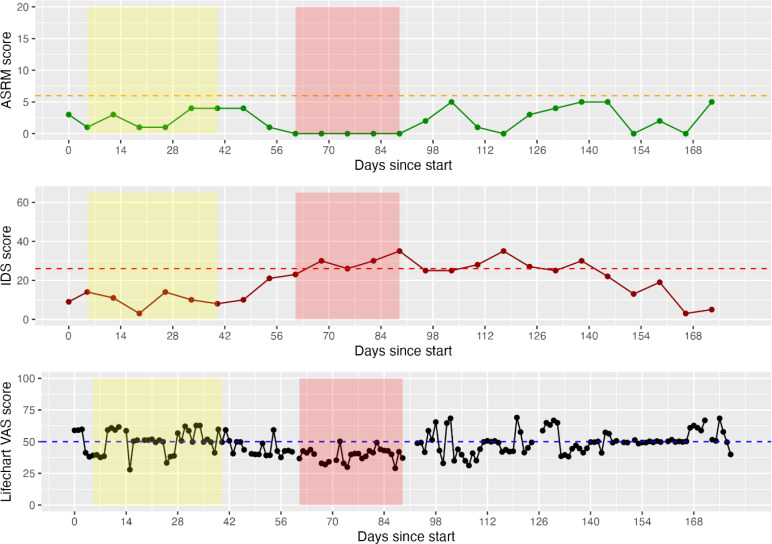
Weekly scores of subject 9

**Figure B6b:**
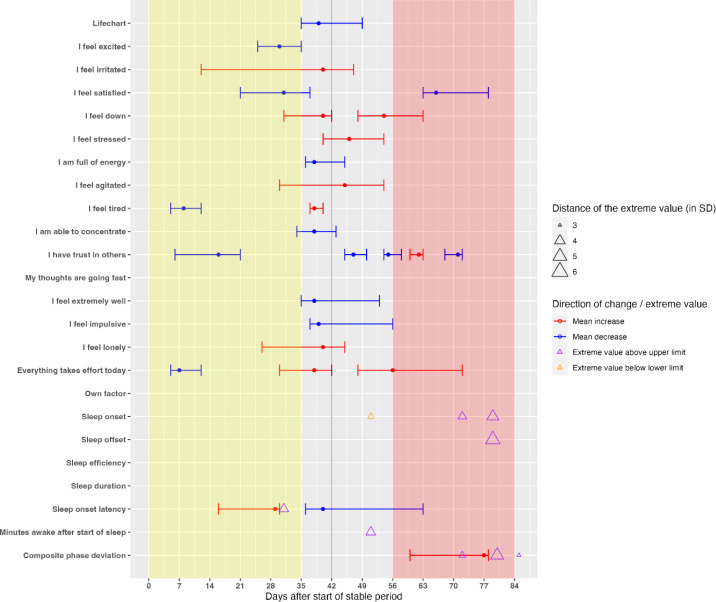
Change points and extreme values of subject 9

**Figure B7a:**
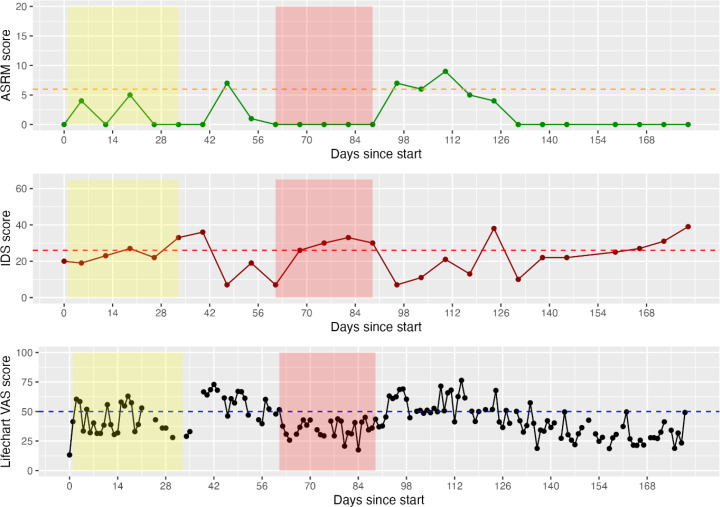
Weekly scores of subject 11

**Figure B7b:**
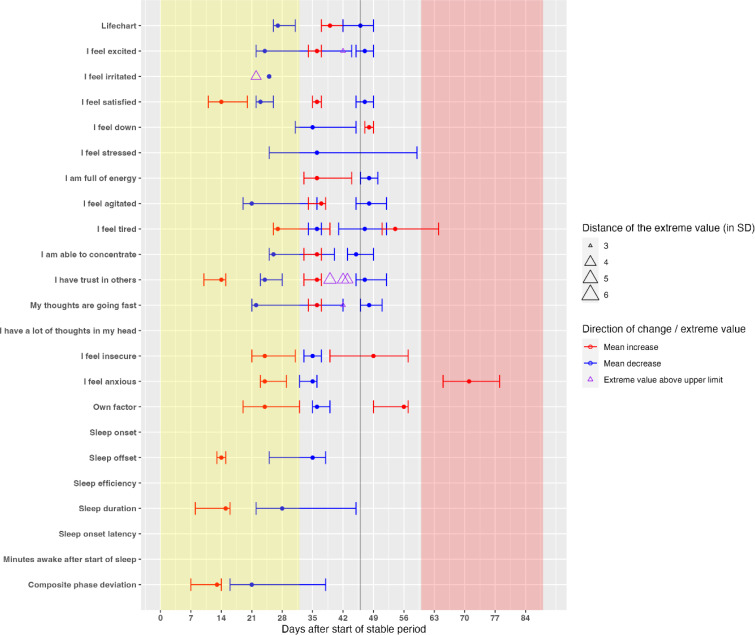
Change points and extreme values of subject 11

**Figure B8a:**
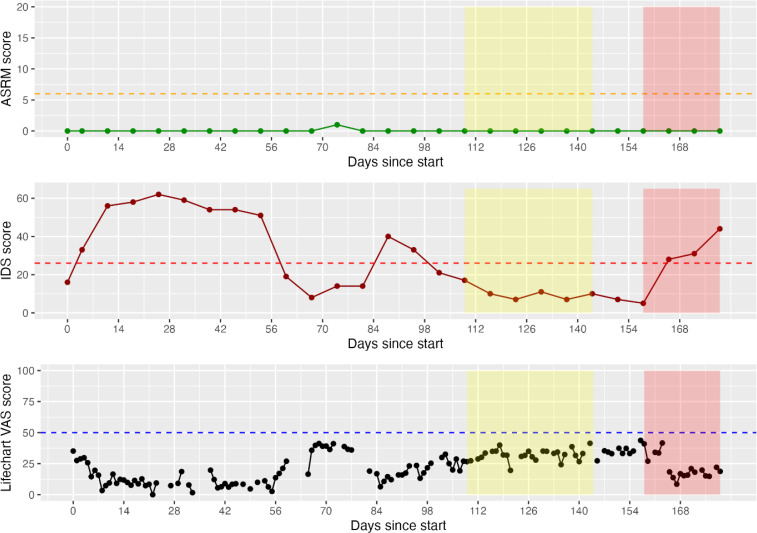
Weekly scores of subject 15

**Figure B8b:**
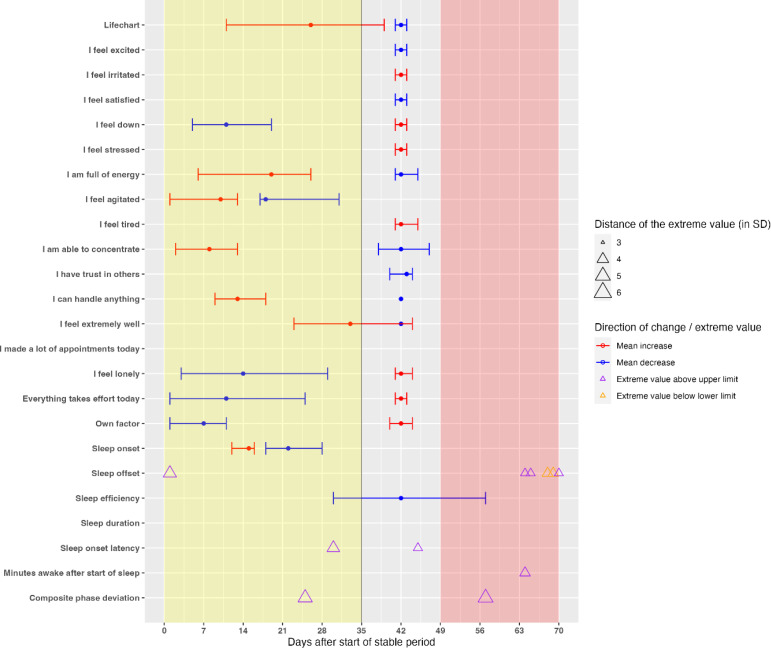
Change points and extreme values of subject 15

## Data Availability

The data are not stored in a public repository due to restrictions related to the European Law regarding General Data Protection Regulation (GDPR), the sensitivity of the data, and the restrictions in the provided informed consent.

## Action editor

Lars-Gunnar Lundh served as action editor for this article.

## Open access

This article is distributed under the terms of the Creative Commons Attribution 4.0 International License (http://creativecommons.org/licenses/by/4.0/), which permits unrestricted use, distribution, and reproduction in any medium, provided you give appropriate credit to the original author(s) and the source, provide a link to the Creative Commons license, and indicate if changes were made.

## Appendix A

**Table A1:** Daily Ecological Momentary Assessment items

**General items**
1. I feel excited
2. I feel irritated
3. I feel satisfied
4. I feel down
5. I feel stressed
6. I am full of energy
7. I feel agitated
8. I feel tired
9. I am able to concentrate
10. I have trust in others
***Mania items* ***
11. My thoughts are going fast
12. I have a lot of thoughts in my head
13. I can handle anything
14. I feel extremely well
15. I made a lot of appointments today
16. I talk a lot today
17. I feel impulsive
***Depression items* ****
18. I feel insecure
19. I feel lonely
20. I feel apathetic
21. I feel anxious
22. I feel guilt
23. I spend a lot of time in bed today
24. Everything takes effort today
25. I worry a lot
***Extra* *****
26. “Own factor”

*Note:*

* Patients were allowed to pick a maximum of three items,

** Patients were allowed to pick a maximum of two items,

*** Patients were allowed to set their own item

## Appendix B

**Figures B1a-B8b**

*Note:* Yellow bar = the stable period, pink bar = the depressive episode, green bar = the manic episode, ASRM score = Altman Self Rating Scale for Manic symptoms, IDS-SR = Inventory of Depressive Symptomatology – Self Rating, days since start = days since start of the study, black vertical line indicated the start of the transition phase.
